# Characteristic postmortem computed tomography findings of ingestion of benzine

**DOI:** 10.1259/bjrcr.20200212

**Published:** 2021-01-15

**Authors:** Hiroyuki Tokue, Yoshihiko Kominato, Rie Sano, Yoichiro Takahashi, Akira Hayakawa, Haruki Fukuda, Azusa Tokue, Yoshito Tsushima

**Affiliations:** 1 Department of Diagnostic and Interventional Radiology, Gunma University Hospital, Gunma, Japan; 2 Department of Legal Medicine, Gunma University, Graduate School of Medicine, Gunma, Japan

## Abstract

There are some reports investigating the cause of death by examining the contents of the stomach and duodenum using postmortem computed tomography, but most of these have been based on radiopaque contents. Here, we report a case of suicide after ingesting a large amount of benzine. Although the gastric contents were radiolucent, the characteristic postmortem computed tomography imaging findings helped to determine the cause of death.

## Introduction

Postmortem computed tomography (PMCT) has been recognized as a supporting diagnostic tool in forensic medicine over the course of the past decade. One of the advantages of PMCT is that it can screen the whole body in a short time. However, gastrointestinal (GI) tract fluid is often a nonspecific finding. The GI density varies with numerous factors such as blood, certain medicines (particularly laxatives), oral contrast medium, and food, among others.^1^ On the other hand, when there is a hyperdense substance in the stomach on PMCT of a case of possible drug addiction, toxicological analysis of the blood and stomach contents may help determine the cause of death. There are some reports that have helped to investigate the cause of death by examining the contents of the stomach and duodenum using PMCT, but most of those have been based on radiopaque contents.^2–4^ Here, we report a case in which characteristic PMCT imaging findings helped identify the cause of death, despite the radiolucent gastric contents.

## Clinical presentation

A 36-year-old male with a history of depression was found dead in a car. When the cadaver was found, no traumatic findings were found throughout his body, but his mouth smelled of an organic solvent. There was also a suicide note and a bottle of benzine beside him. *Toxicology examination of his urine was done and illegal drugs were not detected. Estimated time after death was about 24 h* from the condition of the cadaver. Death by suicide was strongly suspected, and PMCT was performed to investigate the cause of death.

## Investigation

**Figure 1.**
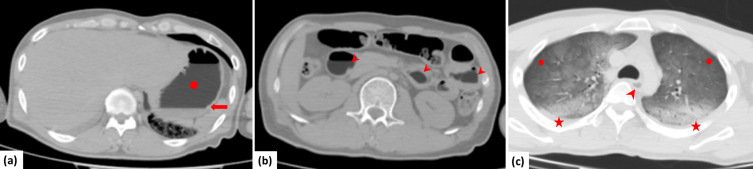
PMCT of a male who died due to benzine poisoning [(**a, b**) window width/window level = 520/–150, (**c**) window width/window level = 1500/–500]. (**a**) PMCT showing two separate fluid layers in the stomach (arrow). The upper layer showed a low density (circle). (**b**) A similar low-density fluid was observed in the duodenum and jejunum (arrowheads). (**c**) Low-density fluid was observed in the esophagus (arrow). Bilateral chemical pneumonia (circles) and postmortem hypostasis (stars) were also observed. The CT number of the low-density fluid was approximately −230 Hounsfield units. PMCT, postmortem computed tomography.

**Figure 2.**
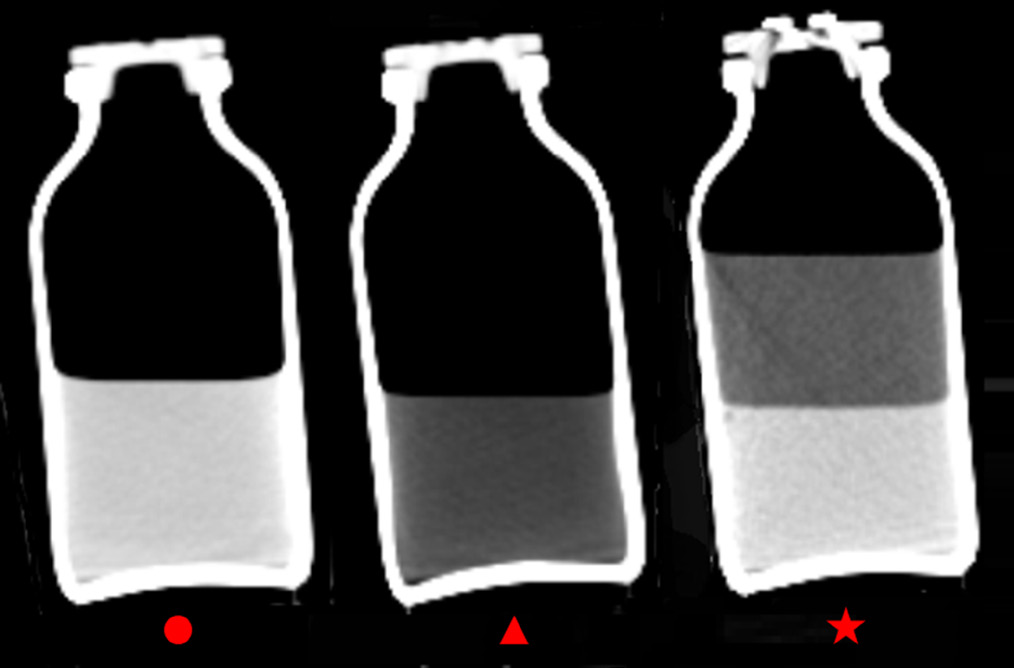
CT of water alone (circle), benzine alone (tringle), and a liquid mixed with benzine and water (star) [window width/window level = 520/–150]. Two separate fluid layers were observed in a mixture of benzine and water, with benzine floating on top of the water. The CT number of benzine was approximately −230 Hounsfield units.

PMCT revealed two separate fluid layers in the stomach. The upper layer had a low density (Figure 1a). Similar low-density fluid was observed in the jejunum, duodenum, and esophagus (Figure 1b and c). The CT number of the low-density fluid in the stomach, jejunum, duodenum, and esophagus was approximately −230 Hounsfield units. Chemical pneumonia was also suspected in both lungs (Figure 1c). No other trauma or organ damage was observed on PMCT. We suspected death from benzine poisoning based on the situation when the cadaver was discovered and PMCT findings. Next, we performed CT of benzine alone and a liquid mixed with benzine and water to confirm reproducibility (Figure 2). The CT revealed very similar characteristic imaging findings to the GI contents seen on PMCT. As a result of comprehensively judging these findings, we decided that the cause of death was benzine poisoning. An autopsy was not performed.

## Discussion

Even though the gold-standard for postmortem forensic assessment is forensic autopsy, PMCT has been used to obtain imaging data instead of or in addition to autopsy in suspicious forensic cases over the last decade. There are some reports that PMCT of high-density gastric contents was useful in evaluating the cause of death in those who have overdosed on medicines.^2^ Medicines containing bromine (*e.g.* bromovalerylurea) are known as radiopaque drugs. In addition, chloral hydrate, heavy metals, iron salts, phenothiazine, and slow-release preparations are also radiopaque medicines.^2,3^ A case of organic mercury poisoning showed hyperdensity along the GI walls.^4^ However, the food residue remaining in the stomach sometimes appears as a hyperdensity and may be difficult to differentiate from a drug.

Benzine is a spirit obtained from petroleum and used as a solvent or fuel. Benzine is a clear, liquid, petroleum-based chemical that has a sweet smell and is readily available commercially. Benzine poisoning occurs when someone swallows, breathes in, or touches benzine. Benzine is a member of a class of compounds known as hydrocarbons. The seriousness of poisoning caused by benzine depends on the amount, route, and duration of exposure, as well as the age and preexisting medical condition of the exposed person. The estimated oral lethal dose for humans is known to be 0.5–5 g/kg.^5^ Based on the remaining amount of the bottle and the measurement of CT images, the estimated oral dose in this case was about 250 cc or more. If benzine enters the trachea, it is likely to cause chemical pneumonitis. Benzine dissolves only slightly in water and floats on top of water. In our case, it is thought that the two separate fluid layers observed in the contents of the stomach on PMCT reflects this. The CT number of petroleum-based chemicals is considered to be negative but may vary depending on impurities. In our case, we examined the CT number of benzine and confirmed that it was consistent with that of the GI contents, and strongly suspected that it reflected benzine poisoning.

There has been no report that the radiolucent contents of the GI tract on PMCT were useful in investigating the cause of death, and this is the first report of the characteristic imaging findings of oral consumption of benzine on PMCT. However, the decision to perform a medicine analysis and autopsy should be made based on all available information, including the circumstances of death.

In conclusion, PMCT may be useful in diagnosing death from poisoning even if the GI contents are radiolucent, and it is necessary to know the imaging findings for radiolucent substances that show characteristic PMCT images.

## Learning points

1. When there is a hyperdense substance in the stomach on PMCT of a case of possible drug addiction, toxicological analysis of the blood and stomach contents may help determine the cause of death.
2. We report a case in which characteristic PMCT imaging findings helped identify the cause of death, despite the radiolucent gastric contents.
